# Application of Optogenetics in Neurodegenerative Diseases

**DOI:** 10.1007/s10571-024-01486-1

**Published:** 2024-07-26

**Authors:** Qian Zhang, Tianjiao Li, Mengying Xu, Binish Islam, Jianwu Wang

**Affiliations:** Xiangya School of Public Health, Central South University, Changsha, 410078 Hunan People’s Republic of China

**Keywords:** Optogenetics, Neurodegenerative diseases, Neuron, Opsin, Electrophysiology

## Abstract

Optogenetics, a revolutionary technique integrating optical and genetic methodologies, offers unparalleled precision in spatial targeting and temporal resolution for cellular control. This approach enables the selective manipulation of specific neuronal populations, inducing subtle electrical changes that significantly impact complex neural circuitry. As optogenetics precisely targets and modulates neuronal activity, it holds the potential for significant breakthroughs in understanding and potentially altering the course of neurodegenerative diseases, characterized by selective neuronal loss leading to functional deficits within the nervous system. The integration of optogenetics into neurodegenerative disease research has significantly advanced in the field, offering new insights and paving the way for innovative treatment strategies. Its application in clinical settings, although still in the nascent stages, suggests a promising future for addressing some of the most challenging aspects of neurodegenerative disorders. In this review, we provide a comprehensive overview of these research undertakings.

## Introduction

Neurodegenerative diseases (NDDs) are a class of pathological conditions characterized by the insidious and progressive degeneration of neuronal structure and function, leading to notable cognitive and motor impairments. NDDs encompasses a variety of disorders, each selectively targeting distinct neuronal populations and manifesting unique pathological hallmarks, predominantly within the central nervous system but occasionally affecting peripheral structures (Wilson et al. 2023).

The prevalence of NDDs has risen in tandem with increased human longevity, presenting a growing public health challenge (Heemels 2016). The gradual neuronal degeneration characteristic of these diseases culminates in significant functional decline, with each disease manifesting a distinct clinical profile reflective of the specific neuronal populations affected (Roselli and Caroni 2015). Addressing the accumulation and deposition of specific pathogenic proteins remains a central challenge in the treatment of NDDs (Crick 1979). Traditional approaches such as medicine or drugs have primarily focused on mitigating symptomatic aspects of these diseases or slowing their progression. However, the advent of more targeted molecular and genetic techniques offers potential avenues for more precise therapeutic interventions targeting the underlying pathophysiological mechanisms specific to each disorder.

Over the past few decades, traditional genetic methods have been instrumental in unraveling the complex etiology and pathogenesis of NDDs. The use of genome-wide association studies has been pivotal in this process, identifying disease genes and risk loci in complex forms of NDDs and revealing the involvement of multiple genes and pathways (Nalls et al. 2019; Andrews et al. 2020). With a deeper understanding of the genetic underpinnings of NDDs, there is a growing need for more precise tools to study neuronal function and dysfunction. Optogenetics, emerging from this need, combines genetic engineering with optical technology to control and monitor the activities of neurons in real time. This technique involves the use of light-sensitive proteins, or opsins, which are introduced into specific neurons via genetic engineering methods. This innovative approach has revolutionized the study of NDDs by enabling precise manipulation and observation of neural circuits (Packer et al. 2013; Shemesh et al. 2017). It has allowed researchers to mimic disease processes, understand the role of specific genes in neuronal function, and explore potential therapeutic targets in a way that was not possible with traditional genetic methods alone.

In this review, we have summarized the methodologies of optogenetic techniques and their applications in NDDs, aiming to provide valuable insights into future research and therapeutic strategies.

## Comparison of Optogenetic Techniques and Other Neural Modulation Methods

**Fig. 1:**
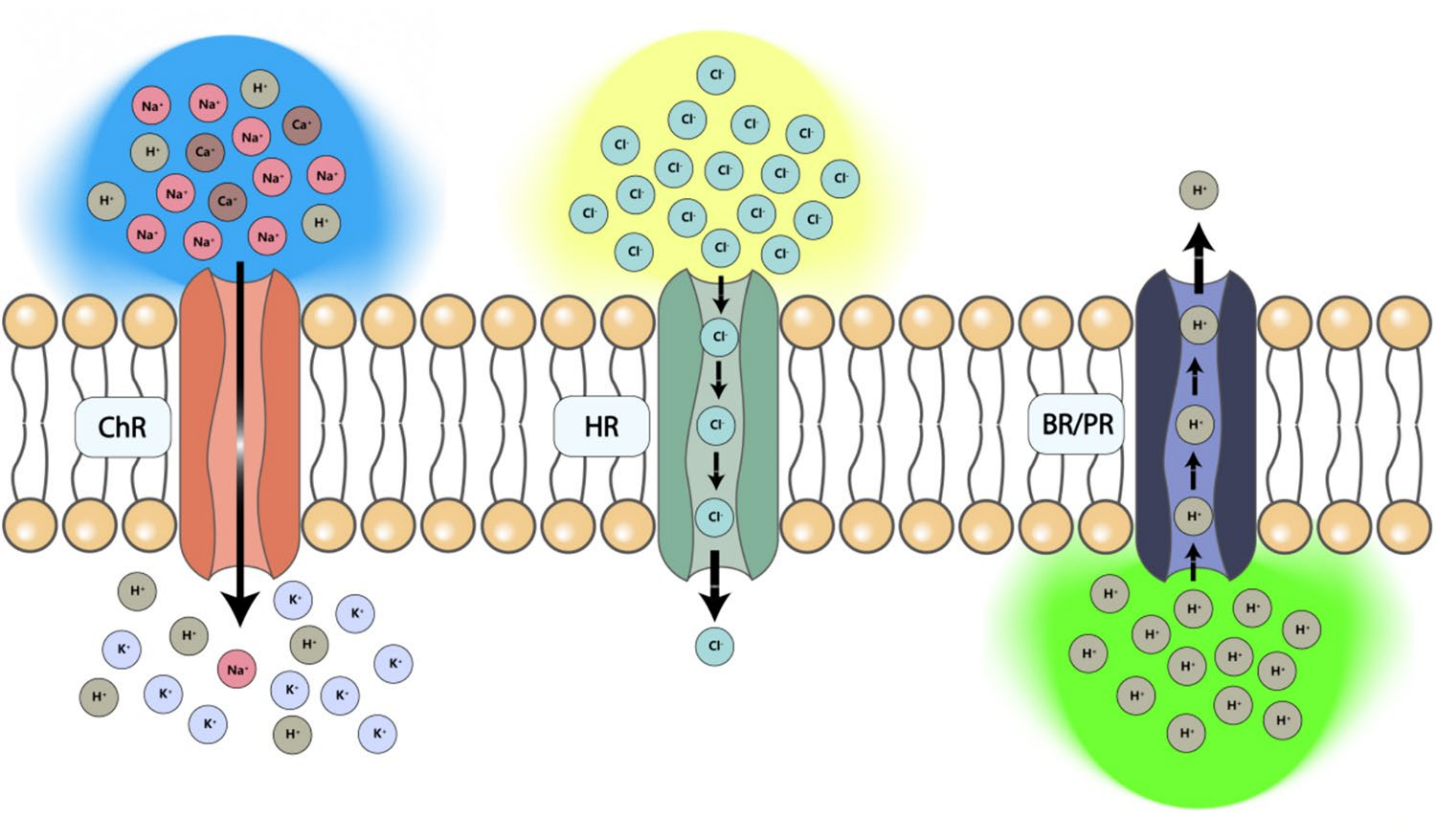
The principles of optogenetics

Optogenetics, an innovative fusion of optics and genetics for cellular control, was developed in the early 2000s by Karl Deisseroth and Ed Boyden. In 2005, Professor Edward S. Boyden inserted the algal protein channel rhodopsin-2 (ChR2) into nerve cells to modulate excitatory synaptic transmission on the millisecond timescale (Boyden et al. 2005). In addition to ChR2, other genetically encoded light-sensitive protein such as halorhodopsins (HR), bacteriorhodopsins (BR) and Proteorhodopsin (PR) as shown in Fig. 1 have been employed for optogenetics by allowing for the inhibition or alteration of specific physiological functions, thus providing direct insights into cellular mechanisms that underlie various physiological processes (Yizhar et al. 2011; Tan et al. 2017). This method employs various light-sensitive proteins to manipulate cells, especially neurons, offering a precise, noninvasive approach to exploring neural circuits, significantly advancing our understanding of brain functions and disorders (Josselyn 2018).

Optogenetics employs photosensitive proteins like ChR are expressed in specific cells. These proteins, when exposed to specific wavelengths of light, activate different ion channels. On the left is ChR, which allows cation influx into the cell upon activation by blue light, causing depolarization. In the center is HR, which pumps chloride ions into the cell under yellow light activation, leading to hyperpolarization. On the right are BR/PR, which pump protons when activated by light, affecting the ion balance within the cell. These photosensitive proteins regulate cellular bioactivity by controlling the flow of specific ions.

**Fig. 2:**
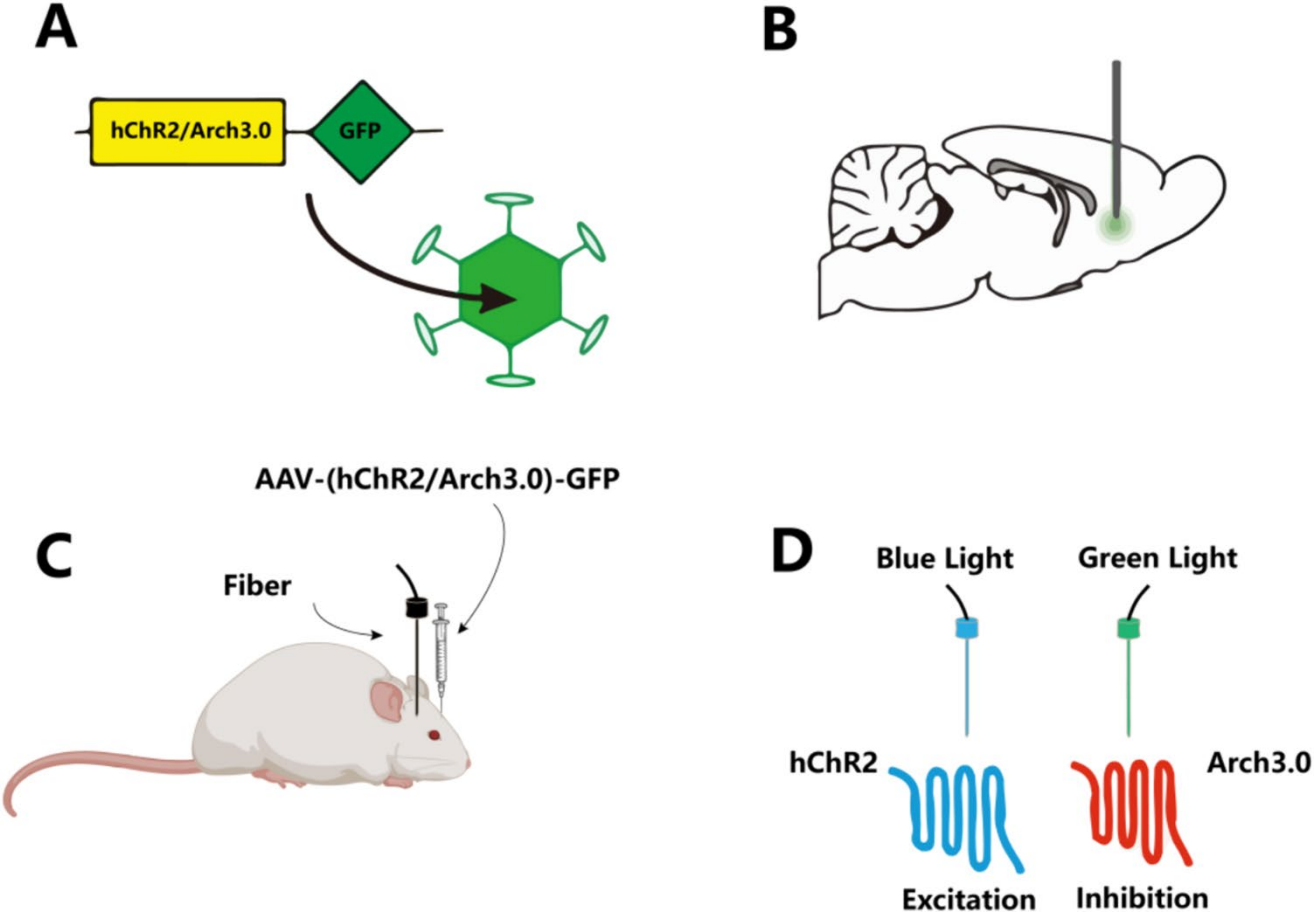
In vivo application process of optogenetics. **A** The use of an AAV vector to deliver genes encoding hChR2 or Arch3.0 fused with GFP into target cells. **B** Shows the injection of the AAV vector into a specific region of an experimental animal’s (such as a mouse) brain to achieve localized expression. **C** The transmission of specific wavelengths of light via an optical fiber to the infected brain area, activating or inhibiting targeted neural cell. **D** The response characteristics of the expressed proteins to different light wavelengths: hChR2, which causes cellular excitation under blue light activation, and Arch3.0, which results in cellular inhibition under the green lighting conditions

The predominant method of optogenetic modulation involves the delivery of genes encoding light-sensitive proteins to target cells via viral vectors. Subsequently, these target cells synthesize light-sensitive proteins and integrate them into their cellular membranes. Upon verification of successful expression of these light-sensitive proteins, the target cells are exposed to light of specific wavelengths (Bernstein and Boyden 2011; Govorunova and Koppel 2016). This exposure induces a conformational change in the proteins, leading to either excitation or inhibition of the cells. These cellular responses can subsequently be monitored using electrophysiological or imaging techniques (Fig. 2). In this process, the selection and application of the appropriate photosensitive protein is the key step. Currently commonly used photosensitive protein includes depolarizing and hyperpolarizing variants, as well as modulators of G-protein-coupled intracellular signaling. Depolarizing tools, like ChR2, open cation channels in response to blue light, leading to depolarization of the cell membrane. On the other hand, hyperpolarizing opsins like Halorhodopsin (NpHR) pump chloride ions in response to yellow light, leading to hyperpolarization (Ferenczi et al. 2019).

In addition to these common photosensitive proteins, emerging proteins such as cytochrome 2(Cry2) has been employed. Cry2 originally identified as a blue light photoreceptor, light-induced aggregation of Cry2-containing peptides uses Cry2 fused to proteins or peptides. When exposed to blue light, these constructs quickly aggregate in cells (Jiang et al. 2021). This technique allows precise control of protein functions and is used to study biological processes, such as protein–protein interactions and the effects of protein clustering on cell functions. It is particularly useful for researching dynamic cellular processes like neurodegeneration. Recently, a novel protein PhoCl has been developed. PhoCl is described as a green-to-red photoconvertible protein that undergoes photocleavage upon activation by near-ultraviolet light, effectively splitting and releasing fused target molecules from the Golgi apparatus (Table 1). This unique capability of PhoCl allows for the controlled release and manipulation of protein functions within live cells, which is crucial for precise studies of cellular processes and the development of therapeutic applications (Kashyap et al. 2024).

**Table 1:** Common photosensitive protein name, category, excitation wavelength, and functional characteristics

Category	Opsin	Excitation wavelength	Characteristics
Activating	hChR2(H134R)	470 nm	A mutant of the most commonly used ChR2; the channel switching speed is also twice as slow as ChR2
	hChR2(E123T/T159C)	470 nm	A mutated ChR2 with a larger photoelectric current and faster kinetics, suitable for high-frequency activation
	Achieve(E163A/T199C)	470 nm	A hybrid of ChR1 and ChR2 can be used for high-frequency stimulation
	C1V1(t/t)	560 nm	A hybrid of ChR1 and VChR1 (mutant of ChR), suitable for specific applications
	nChR2(C128S/D156A)	470–490 nm	Significant stability in the activated state, usable for inducing prolonged depolarization
	ChETA	560 nm	E123T mutation allows neurons to fire spikes at 200 Hz under light stimulation, while other ChR2 channel proteins can only reach 40 Hz
	CheRiff	530 nm	Improved light sensitivity, kinetics, and spectral orthogonality
	ChrimsonR	590 nm	A K176R point mutation of the naturally occurring CnChR1 (Chrimson)
	Chronos	530 nm	Naturally occurring ShChR (Chronos)
	ChroME2s	1030 nm	Fast response, capable of high-frequency neuronal activation; activation intensity (inward current) is greater than the commonly used ChrimsonR
	ReaChR	590–630 nm	Composed of ChEF/ChIEF, VChR1, VChR2 with an added L171I mutation
Inhibitory	eNpHR3.0	589 nm	Third-generation light-driven inward chloride pump, improved expression of NpHR on the cell membrane
	Arch	566 nm	Improving the localization and uniform distribution of the photosensitive tool on the cell membrane
	hGtACR1	540 nm	Anion channel mode of channel rhodopsins; usable for rapid and reversible neuronal silencing
	eArch3.0	590 nm	Larger dynamic range; off-target effects exist when applied to the presynaptic terminal
	eOPN3	512 nm	Well tolerated in mammalian neurons and does not cause significant light-dependent physiological changes in neuronal excitability
	QuasAr2	640 nm	Increased brightness and voltage sensitivity, microsecond response time, and does not produce photocurrent
	SwiChRca	475 nm	C1C2 chimera mutation C128A (SwiChRCA) can slow down channel closure, continuously activate chloride ion channels by a single blue light, keeping the cell in a sustained inhibitory state, and close the chloride ion channels after red light irradiation
	Jaws	632 nm	In a red-shifted chloride ion pump, the hyperpolarization current caused by 632-nm light is significantly larger than eHpHR3.0 or ArchT, mainly used for inhibiting target sites with infrared lasers and even noninvasive lighting methods can inhibit Jaw-infected sites
Two-way Regulation	eNPAC2.0	590–620 nm (inhibitory) 448 nm (activating)	Contains a dual cis–trans sequence of NpHR and ChR2 Excitation
	BiPOLES	595/635 nm (activating) 460 nm (inhibitory)	A fusion of blue light-inhibited GtACR2 and red light-activated Chrimson

Moreover, bioluminescent optogenetics is an innovative integration of bioluminescence and optogenetic, eliminating the need for external light sources. This method leverages bioluminescent proteins, such as luciferase, which can produce light through biochemical reactions within the organism (English et al. 2021). This integration opens new avenues for controlling cellular functions more naturally and effectively within live organisms.

Apart from optogenetics, common neuromodulation techniques include deep brain stimulation (DBS), transcranial magnetic stimulation (TMS), and chemogenetics, each with unique advantages and limitations, as detailed in Table 2.

**Table 2:** Neuro-modulation techniques

Technique	Optogenetics	DBS	TMS	Chemogenetics
Method	Delivery of genes encoding light-sensitive proteins via viral vectors; light exposure induces excitation or inhibition	Surgical implantation of electrodes and a generator; modulation of electrical pulses	Noninvasive; induces weak electric currents in brain regions through magnetic pulse alterations	Genetic introduction of receptors/ion channels sensitive to specific compounds; controlled via small molecular compounds
Specificity	Modulate specific neurons	Target specific brain regions	Target specific brain regions	Modulate specific neurons
Applications	Research in neural circuits; potential in neurological disorders	Parkinson’s disease, essential tremor, dystonia, obsessive–compulsive disorder	Psychiatric and neurological conditions; depression	Research in neuronal function; potential in precise neuronal modulation
Advantages	High specificity	Method is mature	Noninvasive	High specificity
Limitations	Invasive	Invasive; lower specificity	Indirect modulation of neurons	Slower response time due to pharmacokinetics

**Fig. 3:**
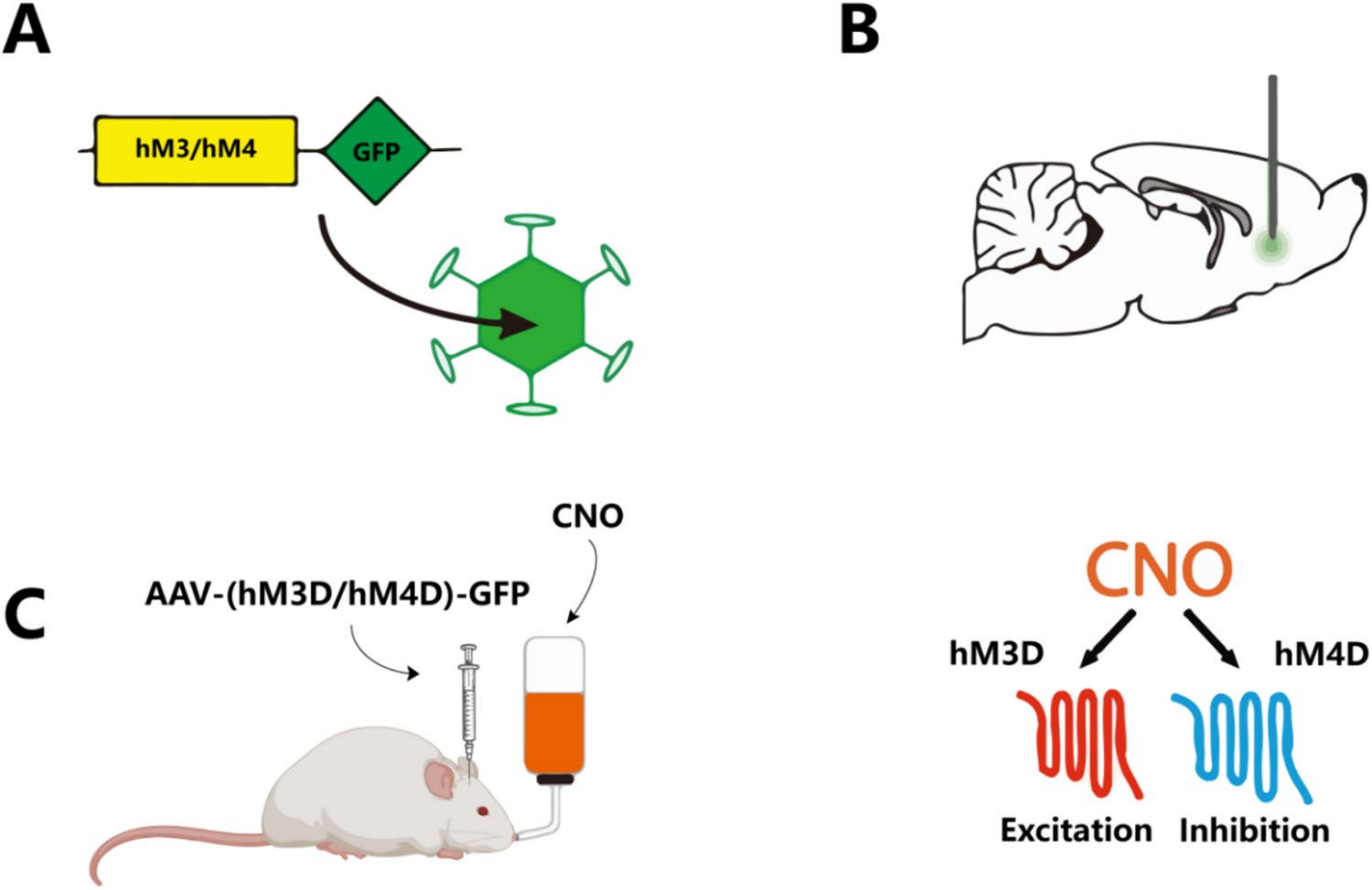
In vivo application process of chemogenetics. **A** A genetic construct containing genes for modified human muscarinic receptors (hM3 and hM4) and GFP. These receptors are engineered to respond exclusively to a synthetic drug called CNO, enabling precise control over neuronal activity. **B** Shows the injection of the AAV vector into a specific region of an experimental animal’s (such as a mouse) brain to achieve localized expression. This targets neurons in the indicated brain region to be genetically modified to express the chemogenetic receptors. **C** CNO has been administered via oral ingestion. This method allows the drug to reach the brain and activate or inhibit neurons that express hM3 or hM4 receptors. hM3 receptors trigger neuronal excitation when bound by CNO, whereas hM4 receptors result in neuronal inhibition

Chemogenetics is a genetic approach that integrates chemical and genetic techniques to study the function of specific molecules or proteins within cells and organisms. This method introduces receptors or ion channels sensitive to specific small molecular compounds into designated neurons or brain areas via genetic engineering. The most common technique used in chemogenetics is known as designer receptors exclusively activated by designer drugs (DREADDs). In contrast to optogenetics, DREADDs are a system of genetically engineered G protein-coupled receptors that enable precise control of receptor activation or inhibition through specially designed small-molecule drugs, such as clozapine-N-oxide (CNO) (Clark et al. 2022). The application of specific small molecules can precisely activate or inhibit targeted neurons, and normal neuronal activity resumes once the compounds are cleared (Fig. 3). Compared to optogenetics, chemogenetics offers high specificity in modulation and is reversible. However, optogenetics provides a faster response time and temporal precision, as it can be controlled on a millisecond scale. In contrast, the response time of chemogenetics is usually subject to the pharmacokinetics of the compounds, resulting in slower reactions (Song et al. 2022).

**Fig. 4:**
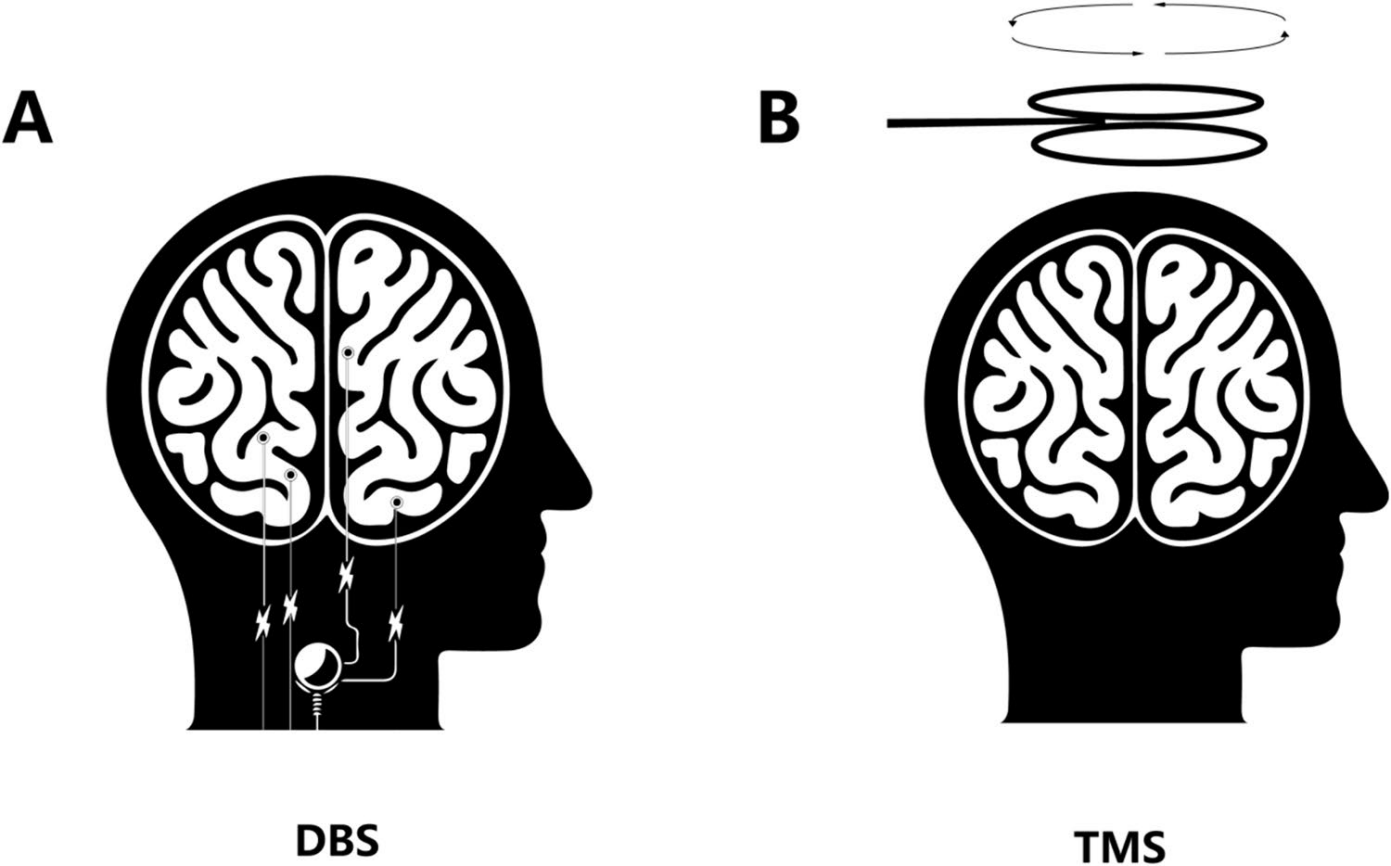
The principles of deep brain stimulation and transcranial magnetic stimulation. **A** DBS uses electrodes implanted in specific brain regions are connected via leads to a pulse generator, delivering electrical impulses to modulate neural activity for treating neurological conditions. It alleviates symptoms by directly stimulating the parts of the brain involved in these diseases. **B** TMS is a non-invasive method that uses a magnetic coil placed on the scalp to generate electrical currents in the underlying brain tissue. TMS offers an alternative to drug therapy and invasive procedures, allowing for targeted stimulation with minimal side effects

DBS is a neuromodulation technique that involves the surgical implantation of electrodes and a generator in specific areas of the brain. This method modulates the intensity, frequency, and duration of electrical pulses, thereby precisely regulating specific brain regions. DBS is commonly used to treat Parkinson’s disease, essential tremor, dystonia, and obsessive–compulsive disorder. Compared to optogenetics, which can modulate specific neurons, DBS generally affects a broader area and therefore exhibits lower specificity (Lozano et al. 2019). (Fig. 4A).

TMS is a noninvasive neuromodulation technique that is considered safer than DBS and optogenetics. It modulates neuronal activity by inducing weak electric currents in specific brain regions through alterations in magnetic pulse intensity, frequency, and duration. High-frequency TMS (> 1 Hz) is typically used to increase neuronal activity, whereas low-frequency TMS (≤ 1 Hz) is employed to suppress neuronal activity. TMS is often used to treat psychiatric and neurological conditions, including depression (Hallett 2007; Fig. 4B).

While each technique possesses unique advantages and a unique scope of application, the high temporal and spatial precision of optogenetics elevates the capacity to manipulate individual neurons to an unprecedented level of control (Tan et al. 2022). This capability not only deepens our understanding of the intricate mechanisms of the brain but also steers the direction for novel interventions in complex neurological disorders, including neurodegenerative diseases. The significance of optogenetics in advancing future neuroscience research and therapeutic approaches is thus prominently underscored.

### Alzheimer’s Disease

Alzheimer’s disease was first identified by German psychiatrist and neuropathologist Alois Alzheimer in 1906. His clinical observations included symptoms, such as memory loss, disorientation, hallucinations, and speech difficulties. Postmortem examination of the patient’s brain revealed distinctive pathological features: abnormal clumps and tangled bundles of fibers. These formations were later identified as Amyloid Plaques and Tau Tangles (Hippius and Neundörfer 2003). Subsequently, extensive neuronal and synaptic loss leading to brain tissue atrophy, neurotransmitter system imbalances, inflammatory responses, and blood‒brain barrier dysfunction were confirmed to be related to the pathogenesis of AD. This understanding has led to the development of several pathogenetic hypotheses, including the amyloid cascade hypothesis, the tau hypothesis (Bloom 2014), and the cholinergic hypothesis (Hampel et al. 2018). In recent years, the metal deposition hypothesis has also gained acceptance in the scientific community (Zatta et al. 2009). These hypotheses highlight the complexity of AD pathology, which collectively leads to cognitive decline, particularly in memory and executive function.

Patients with AD commonly exhibit abnormal processing of amyloid precursor protein (APP), leading to the formation and accumulation of Aβ peptides, which ultimately aggregate into oligomers and plaques. These plaques are neurotoxic, disrupting synaptic connections and signal transmission between neural cells, affecting the release and reception of neurotransmitters, and ultimately impairing neural network functions (Friedrich et al. 2010; Spires-Jones and Hyman 2014; Nativio et al. 2020). Additionally, they activate immune cells, such as microglia, triggering inflammatory responses that further damage neurons and synapses (Griffiths and Grant 2023).

Optogenetic technology, as an effective tool, facilitates the study of this pathological process. Lim et al. used an optogenetic amyloid-β (Aβ) peptide that rapidly oligomerizes under blue light. This technique allows specific control over the timing and extent of Aβ oligomerization. By employing blue light to induce Aβ oligomerization, the metabolic and physical damage caused by Aβ oligomers can be separated from the effects of its mere expression. This differentiation is crucial for understanding the specific impacts of Aβ oligomerization on pathogenesis. The results indicate that light-induced Aβ oligomerization in model organisms such as Drosophila, *C. elegans*, and *Danio rerio* leads to considerable lifespan reduction, behavioral impairments, and metabolic damage. This damage is evidenced by a decrease in mitochondrial numbers and an increase in oxidative stress. In contrast, simply expressing Aβ without oligomerization caused a milder increase in inflammatory markers and other changes, without the extensive physical or morphological damage observed with oligomerization (Lim et al. 2020).

Tau protein is closely associated with the microtubules in neurons. Normally, the tau protein stabilizes microtubule structures within neural cells and is essential for intracellular transport (Lim et al. 2020). In AD, the tau protein undergoes abnormal phosphorylation, losing its normal function and aggregating abnormally to form neurofibrillary tangles, disrupting microtubule function. This impairs the transport system of neurons, affecting cell health and function and thereby advancing neurodegeneration (Zhang et al. 2021). To explore the relationship between tau protein release and neuronal activity, Ismael et al. stimulated Drosophila neurons expressing human tau protein using optogenetics, precisely controlling neuronal activity with blue light stimulation of ChR2 to observe the release of phosphorylated tau during neuronal activation. The results showed a significant increase in the release of hTau 30 min after stimulation, confirming a link between neuronal activity and tau protein (Ismael et al. 2021). Furthermore, Jiang et al. applied light-reactive bacterial Cry2 protein to generate oligomers of tau-Cry2 chimeras in primary cortical neurons in mice with AD and investigated the interactions between tau oligomers, RNA-binding proteins, and N6-methyladenosine RNA. The study showed that light-induced tau oligomers result in neurodegeneration in cortical neuron cultures, as evidenced by reductions in neurite length, aberrant dendritic morphology, increased proapoptotic activity, and elevated lactate dehydrogenase levels, indicating neurotoxicity and impacting protein interactions (Jiang et al. 2021).

The loss of neurons and synapses is a key pathological feature of AD, occurring predominantly in crucial areas for memory processing and cognition, notably in the hippocampus and cerebral cortex (Tzioras et al. 2023). Within the hippocampus, three principal pathways are integral: the perforant pathway, the mossy fiber pathway, and the Schaffer collateral pathway. The perforant pathway connects the entorhinal cortex to the dentate gyrus, the mossy fibers link the dentate gyrus to CA3 neurons, and the Schaffer collaterals connect CA3 to CA1 neurons (Mankin et al. 2015). These synaptic connections are essential for processing and consolidating new memories. Yamamoto et al. focused on the hippocampal perforant pathway, activating neurons with stabilized step function opsin in APP mice to study Aβ plaque accumulation and finding increased deposition in the stimulated area, suggesting that chronic synaptic activity may exacerbate AD pathology (Yamamoto et al. 2015). Additionally, the differential vulnerability of hippocampal CA3-CA1 synapses to Aβ was explored by injecting a viral vector carrying ChR2 into either the left or right CA3 region in mice, enabling real-time observation of changes in synaptic function (Shipton et al. 2022). In addition, ChR2 was delivered to the hippocampal CA3 pyramidal neurons (PNs) of Amyloid Precursor Protein/Presenilin 1 (APP/PS1) mice. In spatially delayed matching to place tasks and spatial objects in place tasks, stimulating CA3 PNs can significantly restore impaired memory in APP/PS1 mice (Yang et al. 2022). Finally, memantine, which is approved for moderate to severe AD, was shown to affect dendritic spine density and functional connectivity in the EC-CA1 pathway, suggesting its potential to enhance cognitive function by improving this pathway (Li et al. 2021).

The dentate gyrus (DG) of the hippocampus is associated with episodic memory and comprises more than 95% of excitable granule neurons and inhibitory GABAergic interneurons (Myers and Scharfman 2009). GABAergic neurons in the DG play a pivotal role in regulating the excitability and plasticity of neural circuits within the hippocampus, influencing memory formation and the overall functioning of the hippocampal network. Using optogenetic technology to activate the DG in Amyloid Precursor Protein Swedish mutation/Presenilin 1 deleted in exon 9 mice, it was found that activation of the DG is sufficient and necessary for memory formation induced by contextual fear conditioning. Direct stimulation of specific engram cells can promote acute memory recall and induce long-term potentiation at perforant path synapses, allowing memories to be recovered in the early stages of AD (Roy et al. 2016; Perusini et al. 2017). In contrast, inhibiting GABAergic interneurons in the DG hampers spatial learning and memory retrieval (Andrews-Zwilling et al. 2012). Activating GABAergic neurons at 2 months of age in APP mice significantly improved their cognitive deficits. However, activating these neurons at a later age, such as 6 months, did not rescue the cognitive impairment in these mice. This finding highlights the potential of early intervention and the specific targeting of GABAergic neurons in the treatment of cognitive deficits associated with AD (Sun et al. 2012). Furthermore, stimulating GABAergic interneurons in the brains of APP/PS1 mice can not only increase gamma and theta power and reduce local field potentials but also activate autophagy and reduce neuroinflammation (Zhang et al. 2020).

The SuM, located in the hypothalamus, is anatomically connected to the hippocampus, particularly the DG. These connections are primarily excitatory, with SuM neurons projecting to the granule cell layer of the DG. This projection plays a significant role in modulating the activity of the DG. AD patients often exhibit adult hippocampal neurogenesis (AHN) impairment. Optogenetic stimulation of the SuM enhances AHN in AD mouse models, and subsequent chemogenetic activation of these SuM-enhanced ABNs can effectively rescue memory and emotional deficits in AD mice (Li et al. 2023).

The integration of optogenetics into AD research has opened new pathways for understanding and potentially treating this complex illness. Through precise control and monitoring of neuronal activity, optogenetics has deepened our comprehension of AD mechanisms at the cellular and molecular levels.

Neural oscillations include alpha, beta, gamma, delta, and theta oscillations, each contributing uniquely to cognitive processing and brain function (Table 3). A decrease in fast rhythm oscillations (alpha, beta, and gamma oscillations) and a general increase in slow rhythm oscillations (delta and theta oscillations) are the most common changes in nerve oscillations in the resting state in AD patients (Jafari et al. 2020). The increase in slow oscillations reflects disrupted brain functional connectivity and cognitive decline, making them markers for tracking disease progression. Optogenetics has enabled researchers to restore slow oscillations in AD models, reducing hyperexcitability and plaque deposition. Researchers achieved this by inducing slow oscillations and restoring their power in APP mice by synchronizing excitatory activity and increasing inhibition in the cortex. This decrease in hyperexcitability was achieved through light activation of ChR2 in pyramidal neurons in layer 5. Specifically, restoring slow oscillations decreased the rate of plaque deposition, possibly due to decreased Aβ production associated with decreased hyperexcitability (Kastanenka et al. 2017).

**Table 3:** Classification and characteristics of hippocampal oscillations

Classification	Alpha oscillations	Beta oscillations	Gamma oscillations	Delta oscillations	Theta oscillations	Slow oscillations	Pathological oscillations
Frequency	8–12 Hz	13–30 Hz	25–140 Hz	1–4 Hz	4–8 Hz	< 1 Hz	Wide range
Property	Physiological						Pathological
Common brain regions	Occipital and parietal regions	Prefrontal lobe	Widely distributed the cerebral cortex	Widely distributed of the cerebral cortex	It is particularly evident in the hippocampus and adjacent regions and is also observed in the cerebral cortex	Widely distributed of the cerebral cortex	Depending on the disease
Characteristics	Mainly appear in a relaxed and eyes-closed state, associated with mental and physical relaxation and attention regulation	Occur in awake and alert states, related to heightened attention and cognitive activities	Related to advanced cognitive functions, such as perception and focused attention, indicating integration of information across brain regions	Primarily occur in deep sleep, associated with physical recovery and memory consolidation	Related to spatial memory and attention regulation	Associated with memory consolidation and replay, predominantly active during rest and non-REM sleep	Abnormal brain electrical activities occurring in neurological diseases, such as epilepsy, potentially disrupt normal brain functions

Optogenetic techniques are commonly applied to gamma and theta oscillations. One study showed that the Aβ load and the power spectral density of gamma oscillations have an inverted U-shaped relationship, which can reflect compensatory effects in the preclinical stage and accelerated pathological changes after decompensation in the later stage (Guan et al. 2022). It has been confirmed that 40-Hz stimulation can significantly reduce the level of Aβ in 5 × Familial Alzheimer’s Disease mice (FAD), APP/PS1, and wild-type mouse models, and the protective effect of gamma stimulation can be extended to other pathogenic proteins. This oscillation can also reduce phosphorylated tau in TauP301S mice (Iaccarino et al. 2016; Singer et al. 2018). Furthermore, Aβ oligomers disrupt theta and gamma oscillations in mice. By expressing ChR2 in somatostatin (SST) and parvalbumin (PV) interneurons in the hippocampus, researchers have stimulated and focused on SST and PV interneurons to explore their roles in theta and gamma oscillations. This study concluded that the deficits in theta and gamma oscillations in AD models could be ameliorated through the optogenetic activation of SST and PV interneurons, respectively (Chung et al. 2020; Park et al. 2020). Similarly, optogenetic activation of ECII_PN_-CA1_PV_ (CA1 parvalbumin) synapses with a theta burst stimulation paradigm was found to effectively interrupt the progression of synaptic decay and improve spatial learning and memory in AD mice (Yang et al. 2018). In addition, after ChR2 is expressed under the CaMKIla promoter to control the pyramidal cells of CA1, optical stimulation of CA1 at the theta frequency can induce robust theta-nested gamma oscillations with a temporal and spatial profile similar to that of CA1 gamma. Cell-attached and whole-cell recordings indicated that excitatory neuron firing slightly preceded interneuron firing within each gamma cycle, suggesting that the intrinsic CA1 gamma oscillation is generated through a pyramidal-interneuron circuit mechanism. Additionally, 40 Hz stimulation of medial septum parvalbumin cells in Parvalbumin-Cre-Positive J20 mice can help in the restoration of low-frequency gamma oscillations, enhance theta-gamma phase-amplitude coupling in the hippocampus, and improve spatial memory (Etter et al. 2019).

By manipulating specific brain circuits and oscillations, optogenetics technology has illuminated the roles of key pathological features, such as amyloid-beta and tau protein, offering new therapeutic avenues. Its application in AD models shows promise for restoring cognitive functions and understanding the intricate interactions that drive Alzheimer’s disease progression. We have summarized the current application of optogenetic technology in NDDs, including AD, with the hope that this technology will facilitate research into mechanisms and drug development (Table 4).

**Table 4:** Overview of the Optogenetics of NNDs

Category	Opsin	Model	Function	References
AD	ChR2	Drosophila neurons	Induce cellular depolarization and trigger tau release	Ismael et al. (2021)
		5 × FAD mice	Activation of SuM to enhance ABNs	Li et al. (2023)
		APP/PS1 mice	Selectively activate GABAergic neurons	Zhang et al. (2020)
		5xFAD	Stimulation FS-PV-interneurons	Iaccarino et al. (2016)
		APP/PS1 mice	Measured functional connection between EC and CA1	Li et al. (2021)
	ChR2 (E123A)	APP Tg mice	Activation of ECII_PN_–CA1_PV_ synapses	Yang et al. (2018)
	ChR2(E123T/T159C)	APP/PS1 mice	Activating previously learned DG memory traces	Perusini et al. (2017)
		APP mice	Drive normal slow oscillations in the brain	Kastanenka et al. (2017)
		APP/PS1 mice	Selectively activate CA3 pyramidal neurons in the hippocampus	Yang et al. (2022)
		Mapt^−/−^ mice	Selectively recruit CA3-CA1 synapses originating in either the left or right CA3	Shipton et al. (2022)
		Aβ_1-42_-injected mice	Manipulate SST and PV interneurons expressing ChR2 in AβO-injected mice	Chung et al. (2020)
	Cry2	SH-SY5Y, N2a, and HEK cells	Induce tau oligomers (Ota-c) and elicit tau phosphorylation, aggregation	Jiang et al. (2021)
	ChETA	J20-APP AD mouse	Activate medial septal parvalbumin neurons at different frequencies	Etter et al. (2019)
	eNpHR3.0	I12b-Cre transgenic mice	Inhibit hilar GABAergic interneuron activity	Andrews-Zwilling et al. (2012)
	oChIEF	3xTg-AD mice	Induction of long-term potentiation (LTP) at perforant path (PP) synapses of DG engram cells	Roy et al. (2016)
	SSFO	APP Tg mice	Selectively stimulate the cortical projection neurons in the lateral entorhinal cortex (LEC)	Yamamoto et al. (2015)
PD	ChR2 (H134R)	D1R-Cre mice	Activation of striatal D1R neurons	Dong et al. (2022)
		6-OHDA lesion rat	Induce dyskinesias in a hemiparkinsonian model of PD in rats	Ledia et al. (2017)
		6-OHDA-lesion mice	The effects of optogenetic PC stimulation on LID	Coutant et al. (2022)
		6-OHDA-lesion mice	Prolonged optogenetic stimulation of dopamine neurons	Malave et al. (2021)
	ChR2 (H134R) and NpHR3.0	6-OHDA lesion mice	Bidirectionally modulate STN neurons and their axonal projections	Yoon et al. (2016)
	ChR2 and eNpHR3.0	AAV2-α-synuclein-induced rat	Rapidly bidirectional control of neurons on the timescale of milliseconds	Moon et al. (2018)
	ChR2 and NpHR	Balb/c mice	Activation or inhibition of grafted cells and host neurons	Tønnesen et al. (2011)
		A53T rat and 6-OHDA-lesion rat	Activation or inhibition of SNpc neurons	Heo et al. (2020)
	ChR2(H134R) and eArch3.0	Pitx2-Cre mice	Excitation and inhibition using both bilateral and unilateral stimulations of the STN	Guillaumin et al. (2021)
	ChR2(H134R) and eNpHR3.0	6-OHDA lesion mice	Activate separately the striatal direct (D1R- expressing) and indirect (D2R-expressing) pathways in a mouse model of PD	Castela et al. (2023)
	Chronos	Dopamine-depleted mice	Identified cellular nodes in the external GPe of the basal ganglia	Spix et al. (2021)
	dR	Drosophila	Allowing for the precise control of mitochondrial function	Imai et al. (2019)
	eNpHR3.0	6-OHDA lesion mice	Modulate in real-time electrophysiological and neurochemical properties of mesencephalic dopaminergic (mesDA) neurons	Steinbeck et al. (2015)
	LMO3	Pitx3 mutation mice	Stimulation of transplanted neuronal precursor cells	Zenchak et al. (2020)
	NpHR	6-OHDA lesion mice	Inactivate the entopeduncular nucleus (EP)	Yoon et al. (2020)
		6-OHDA lesion mice	Inhibit neuronal activity in the STN	Yoon et al. (2014)
HD	ArchT	Drd2-tTA BAC Tg mice	Inhibition and ablation of VLS D2-MSNs	Tsutsui-Kimura et al. (2017)
	ChR2	R6/2 Transgenic Mouse	Activate selectively MSNs to determine their relative contribution to GABA synaptic activity in SNr and GPe neurons	Barry et al. (2018)
		R6/2 Transgenic Mouse	Stimulation to activate iMSNs selectively	Barry et al. (2020)
		R6/2 Transgenic Mouse	Selective activation of GABAergic interneurons	Cepeda et al. (2013)
	ChR2 (H134H)	R6/1 Transgenic mice	Stimulation of the M2 cortex to investigate M2 cortex projection to the SC	Conde-Berriozabal et al. (2023)
		R6/1 Transgenic mice	Induced glutamate release from M2 cortex terminals in the dorsolateral striatum	Fernández-García et al. (2020)
	ChR2 (H134R)	Q175 mouse	Selectively interrogate the functional connectivity of IT and PT cortico-striatal terminals with identified iSPNs and dSPNs	Pancani et al. (2023)
		Q175 mouse	Strong activation of cortico-striatal	Tanimura et al. (2016)
		TH-Cre^+^ rats	Stimulated SNc dopamine neurons during and after rat skilled reaching	Fernández-García et al. (2020)
ALS	ChR2	Drosophila larvae	Increase intracellular calcium level in larval C4da neurons	Park et al. (2020)
	ChR2 (H134R)	iPS-derived muscle cells	Facilitate spatiotemporal control of neural activity and muscle contractions in a micro physiological 3D model of ALS	Osaki et al. (2018)
	OptoTDP43	Drosophila	Light-inducible persistent insoluble species and progressive motor dysfunction	Otte et al. (2020)
	OpTDP-43	Zebrafish	Precise control over the timing and location of opTDP-43 multimerization and aggregation	Asakawa et al. (2020)
	Opto-G3BP1	iPSCs cells	Experimental control of SGs in living cells in the absence of exogenous stressors	Zhang et al. (2019)
	Cry2olig-TDP-43	Human cortical-like neuronal cells	Induce TDP-43 proteinopathy with blue light and examine the mechanisms that drive the formation of intracellular inclusions	Mann et al. (2019)

### Parkinson’s Disease

Parkinson’s disease (PD), initially described by Dr. James Parkinson in 1817, is a progressive neurological disorder characterized by a spectrum of symptoms, both motor and non-motor. The primary motor manifestations, such as bradykinesia, rigidity, tremors, and postural instability, are predominantly attributed to the degeneration of dopaminergic neurons within the substantia nigra pars compacta (Balestrino and Schapira 2020). Complementing these motor symptoms, PD patients frequently exhibit a range of non-motor symptoms, including but not limited to cognitive deficits, mood disorders, sleep disruptions, autonomic dysfunction, and sensory disturbances (Schapira et al. 2017). Neuropathologically, PD is characterized by the presence of Lewy bodies and intracellular inclusions primarily composed of abnormally folded alpha-synuclein, which play a significant role in the progression of neurodegeneration (Cacabelos 2017). The etiology of PD is multifactorial, involving an interplay between genetic susceptibilities—indicated by mutations in genes such as SNCA, LRRK2, PARK2, PINK1, and DJ-1—and environmental influences, including toxin exposure (Chang and Chen 2020). Additionally, oxidative stress, characterized by an imbalance of reactive oxygen species, and mitochondrial dysfunction are crucial in mediating neuronal death in PD (Subramaniam and Chesselet 2013). Neuroinflammation, especially characterized by the activation of microglia and subsequent release of proinflammatory mediators, is also a significant contributor to disease progression (Heidari et al. 2022). In addition, emerging theories suggest the possibility of a prion-like mechanism in the spread of alpha-synuclein pathology, highlighting a systemic aspect of the disease (Butler et al. 2022). This complex interrelation of genetic, environmental, and pathological factors elucidates the intricate nature of PD.

Striatal dopamine D1 receptor neurons are crucially implicated in PD pathophysiology, primarily due to dopaminergic neuronal loss in the substantia nigra. This loss leads to a significant reduction in striatal dopamine levels, disrupting the balance between the direct and indirect pathways in the basal ganglia circuitry. D1 receptor neurons, which are integral to the direct pathway, facilitate movement initiation and execution. In PD, the diminished dopamine supply results in the underactivation of these neurons, contributing to cardinal motor symptoms, such as bradykinesia and rigidity (Barroso-Chinea et al. 2015). Contemporary research and therapeutic strategies are focusing on rectifying this imbalance. Pharmacological interventions include the use of dopaminergic agents, such as levodopa, which aim to replenish dopamine deficiency and thereby enhance D1 receptor activation (Aubert et al. 2005). However, long-term levodopa usage can lead to complications, such as levodopa-induced dyskinesias, which are attributed to the overstimulation of D1 receptors. Optogenetic technology has the potential to elucidate the mechanisms of LID and mitigate its severity.

Selective activation of D1 receptor-expressing medium spiny neurons (D1-MSNs) in the direct pathway of the striatum in a mouse model of PD was found to induce dyskinesias, both in dopamine-depleted and intact animals (Castela et al. 2023). Similarly, simultaneous optical activation of medium spiny neurons of both direct and indirect striatal pathways in dopamine-depleted rats induced involuntary movements resembling LID (Ledia et al. 2017). This was evidenced by the fact that optogenetic activation of D1-MSNs in the direct pathway led to the production of dyskinesia-like movements even in the absence of levodopa. The activation of D1-MSNs is causally involved in the development of LID (Girasole et al. 2018). Daily short-term light stimulation of Purkinje cells with ChR2 in classical LID mice has been shown to inhibit and prevent the development of LID and reverse the discharge of abnormal neurons in the cerebellar nucleus, motor cortex, and thalamus, thus restoring extensive motor function and possibly explaining why cerebellar stimulation alleviates LID. These effects are related to the reversal of plasticity in D1 striatal neurons and the normalization of the overexpression of FosB, a transcription factor causally linked to LID (Coutant et al. 2022). Similarly, longer pulse optical stimulation of striatal cholinergic neurons decreased L-DOPA-induced AIMs in a 6-OHDA lesion mouse model of PD. This effect is associated with an increase in c-Fos expression in ChAT neurons, suggesting that changes in the activity of these neurons may be involved in the modulation of LIDs (Bordia et al. 2016). However, long-term blue light stimulation decreased sonic hedgehog activity and induced LID-like movements in mice expressing ChR2 in dopamine neurons without levodopa (Malave et al. 2021). These findings highlight the need for the cautious application of optogenetics, considering its potential adverse effects. Nevertheless, its judicious use could offer novel treatment avenues for PD. Additionally, optogenetics confirmed that the activation of striatal dopamine D1 receptor neurons can induce an immediate transition from non-rapid eye movement sleep to awakening in mice, providing insights into potential treatments for sleep disorders in PD patients. This finding underscores the multifaceted role of D1 receptor neurons in PD and the promise of optogenetics in offering more nuanced and targeted therapeutic approaches (Dong et al. 2022).

Apart from pharmacotherapy, deep brain stimulation (DBS), stem cell therapy (SCT), surgery, and gene therapy are effective treatments for PD used in clinical settings.

DBS, which has high therapeutic efficacy in PD, is well established but is accompanied by significant side effects. Its lack of specificity for neuronal circuits and rapid attenuation of effects after stimulation interruption limit its long-term efficacy (Histed et al. 2009). The use of optogenetics can identify certain cellular nodules. Researchers have used optogenetics to identify and differentiate between two specific types of neurons in the external globus pallidus (GPe) of the basal ganglia. These were parvalbumin-expressing (PV-GPe) neurons and lim–homeobox–6–expressing (Lhx6-GPe) neurons. By activating PV-GPe neurons while simultaneously inhibiting Lhx6-GPe neurons in dopamine-depleted mice, researchers were able to demonstrate that targeted interventions at these cellular nodes could not only ameliorate circuit dysfunction but also restore movement for several hours after stimulation. In this way, replicating the cell type-specific effects observed with optogenetics using electrical stimulation led to the development of ‘burst DBS,’ a novel form of DBS that mimicked the optogenetic effects by exciting and inhibiting the same neuron types as identified in the optogenetic part of the study. Burst DBS was shown to provide long-lasting therapeutic benefits that far exceeded those induced by conventional DBS. (Spix et al. 2021). The main therapeutic targets for DBS are the subthalamic nucleus (STN) and globus pallidus nucleus (GPI) (Obeso et al. 2001). Optogenetic manipulation in mice, through the activation of STN neurons by ChR2 (H134R) and the inhibition of GPI neurons by eArch3.0 and NpHR, can differentially influence contralateral forelimb dyskinesia and potentially modulate LID symptoms (Yoon et al. 2014, 2016, 2020; Moon et al. 2018; Guillaumin et al. 2021). Furthermore, DBS is also beneficial for relieving non-motor symptoms. The symptoms of patients with PD who experience musculoskeletal and dystonic pain can be relieved by STN stimulation. An optogenetic technique confirmed that the inhibition of overactive STN neurons could significantly reduce pain hypersensitivity. Conversely, induced hyperactivity of STN neurons can cause pain hypersensitivity (Luan et al. 2020). These findings provide a rationale for the effectiveness of DBS and indicate that optogenetics is expected to be a viable complementary option.

SCT for PD treatment is anticipated to become a new treatment modality in which optogenetics can play a valuable role in exploring graft function and enhancing therapeutic effects (Lindvall 2016; Barker 2019). Tønnesen et al. combined patch-clamp recordings with optogenetic tools to investigate the integration of stem cell-derived dopaminergic (DA) neurons grafted into an in vitro PD model. These findings demonstrate that these grafted neurons in the striatum exhibit mature properties akin to those of DAergic neurons in the substantia nigra. Furthermore, the study revealed complex bidirectional synaptic interactions between the grafted neurons and host cells, highlighting significant synaptic connectivity within the graft (Tønnesen et al. 2011). Additionally, PD mice with motor deficits can recover from grafted mesencephalic dopaminergic neurons. However, the light-induced selective silencing of graft activity can cause motor defects to occur rapidly and reversibly, providing evidence for the effectiveness of SCT (Steinbeck et al. 2015). Zenchak et al. explored bioluminescence-driven optogenetic activation for treating PD. They developed a neural stem cell line expressing luminopsin 3 (LMO3), which is activated upon light emission induced by coelenterazine. Transplanting these cells into the striatum of PD model mice with the Pitx3ak mutation significantly improved motor skills upon coelenterazine stimulation. This method, which integrates targeted stimulation with grafts, showed that early stimulation can lead to lasting reductions in motor deficits, highlighting a promising direction for Parkinson’s disease treatment (Zenchak et al. 2020).

Complex I, also known as NADH:ubiquinone oxidoreductase, is a crucial enzyme in the mitochondrial electron transport chain and plays a pivotal role in cellular energy production (Titov et al. 2016). In PD patients, there is a notable reduction in complex I activity, particularly in the substantia nigra, the brain region significantly impacted by the disease. This deficiency hinders the normal oxidative phosphorylation process, leading to reduced ATP generation and enhanced reactive oxygen species (ROS) production. The consequent decrease in ATP production critically affects the survival and function of neurons, especially the energy-intensive dopaminergic neurons in the substantia nigra. These neurons, which have extensive axonal and dendritic networks and high electrical activity, are particularly susceptible to energy deficits (Lv et al. 2022). Additionally, increased oxidative stress, exacerbated by the inherent high oxidative metabolic rate of neurons and the oxidative by-products of dopamine metabolism, contributes to the progressive neuronal degeneration observed in PD. This oxidative damage further impairs mitochondrial function, creating a detrimental cycle of mitochondrial damage and ROS production, which not only accelerates neuronal loss but also influences other pathological hallmarks of PD, such as α-synuclein aggregation and Lewy body formation (Subramaniam and Chesselet 2013).

To counteract these mitochondrial dysfunctions, therapeutic strategies currently focus on enhancing mitochondrial function and reducing oxidative stress. These include the use of coenzyme Q10 and mitochondrion-targeted antioxidants and drugs such as PGC-1α activators that promote mitochondrial biogenesis, potentially restoring mitochondrial density and function in dopaminergic neurons (Wu and Frucht 2005; Kuczynska et al. 2021). Advances in gene therapy also show promise for correcting mitochondrial DNA mutations and delivering protective mitochondrial genes (Gorman et al. 2016). Furthermore, optogenetics has identified novel potential targets for PD treatment, emphasizing the improvement of mitochondrial function. One such development is the use of delta-rhodopsin (dR), a light-driven proton transporter, in Drosophila mitochondria. This approach maintains the mitochondrial proton-motive force and membrane potential in a light-dependent manner. The activation of mitochondrion-targeted dRs (mito-dRs) has been shown potential to reverse the reduction in ATP synthesis caused by CHCHD2 deletion by mitigating mitochondrial peroxide production and reducing Ca2^+^-buffering activity in dopaminergic neurons. It also prevents α-synuclein aggregation, reduces dopaminergic neuronal loss and decreases lipid peroxidation in brain tissue, ultimately improving motor behaviors (Imai et al. 2019). These advancements highlight the critical role of mitochondrial health in PD pathogenesis and offer promising avenues for therapeutic intervention.

### Huntington’s Disease

Huntington’s disease (HD) is a neurodegenerative disorder marked by an aberrant CAG expansion in exon 1 of *Huntingtin*, leading to a mutated HTT protein with an abnormally elongated polyglutamine tract (Gu et al. 2022). This mutation results in the accumulation of HTT protein aggregates in both the nucleus and cytoplasm of neurons, particularly in the striatum, disrupting cellular homeostasis and leading to neuronal death (Chen et al. 2022). These pathological changes are closely associated with the characteristic symptoms of HD, such as choreiform movements, which are involuntary, rapid, and jerky motions, and cognitive impairments.

The pathophysiology of HD predominantly initiates with the degeneration of indirect pathway medium spiny projection neurons (iMSNs) that project to the external segments of the external globus pallidus (GPe). This degeneration disrupts the balance within the basal ganglia circuitry, which plays a critical role in coordinating and regulating motor control. As the disease progresses, there is a notable increase in neuronal apoptosis, particularly affecting these iMSNs, which further exacerbates motor and cognitive deficits(Reiner 2004; Perez-Rosello et al. 2019). This underlines the importance of the basal ganglia and its neural pathways in the clinical manifestations of HD.

Optogenetic techniques can aid in understanding neuropathology and support the discovery of potential therapeutic targets (Table 4). In zQ175 heterozygous HD mice, early symptoms were accompanied by increased connections between the intratelencephalic pathway and direct and indirect pathway MSNs rather than between the intratelencephalic pathway and the pyramidal tract. Additionally, it has been confirmed that presynaptic inhibition of extracerebral terminals by striatal cholinergic intermediate neurons decreases (Pancani et al. 2023). Similarly, by detecting alterations in the intrinsic and synaptic properties of SNr and GPe neurons from R6/2 and YAC128 HD mice, it was found that the amplitude of the GABA response of SNr neurons in both mice decreased after optogenetic stimulation at the terminal of the direct pathway. The amplitude and frequency of spontaneous GABAergic synaptic activity induced by terminal iMSNs in HD mice were similar to those observed in the control group. However, the attenuation time of the induced GABA response was significantly longer. The difference between the responses of the two pathway terminals in the SNr and GPe leads to an imbalance in striatal and motor dysfunction (Barry et al. 2018). This conclusion was also verified by Barry et al. and Cepeda et al. who reported that when ChR2 was utilized to selectively activate GABAergic intermediate neurons, the synaptic activity of GABAergic neurons targeting iMSNs increased. Abnormalities in GABAergic nerve transmission may be an important reason for the interruption of cholinergic interneurons (ChIs) in the striatum (Cepeda et al. 2013; Barry et al. 2020). An investigation of the impact of the ChR2 protein on the synaptic characteristics of ChIs in Q175 mice revealed that the boosting influence of thalamic striatal synapses on ChIs decreased and that there was a reorganization of the striatal functional network. In addition, as the disease progressed, there was a decrease in MSNs. Although large cholinergic interneurons (LCIs) avoid this scenario, their ability to release acetylcholine is diminished. In LCIs, the presence of mutant HTT protein as well as morphological and electrophysiological anomalies provide evidence for explaining HD cholinergic deficits (Holley et al. 2015; Tanimura et al. 2016).

The premotor area of the cortex is the cortical input that most affects the striatum in the early stage in HD patients, which is related to motor learning impairment. A recent study conducted on R6/1 HD mice demonstrated the ability to directly modulate neuronal activity using blue light stimulation targeted at ChR2 expressed in the premotor cortex. This optogenetic activation was precisely synchronized with electrophysiological recordings from a multielectrode array, which captured significant changes in postsynaptic currents within the striatum. Notably, the altered neuronal response to unexpected sensory stimuli, including sudden flashes of light, underscored the disruption of the cortico-striatal pathway, a critical element in motor control and learning. These findings illuminate the underlying neurobiological mechanisms contributing to motor learning deficits observed in the early stages of HD, offering a clearer understanding of the disease’s progression and potential therapeutic targets (Conde-Berriozabal et al. 2023). Surprisingly, frequent blue light stimulation of the M2-dorsolateral striatum in HD mice expressing hChR2 under the CaMKIIa promoter restored motor function and increased synaptic plasticity (Fernández-García et al. 2020). Moreover, the fundamental reason behind impaired motivation in HD patients is widespread dysfunction within various neural circuits across the nervous system. Loss of dopamine receptor type 2-expressing striatal medium spiny neurons (D2-MSNs) in the ventral striatum (VLS) reduces goal-oriented behavior without affecting reward preference or spontaneous behavior. Optogenetic inhibition and ablation of VLS D2-MSNs lead to transient and chronic reductions in goal-oriented behavior, respectively, which confirms that the loss of VLS D2-MSN function is a key node of impaired motivation (Tsutsui-Kimura et al. 2017). Collectively, these studies confirm the potential of optogenetic techniques as effective tools for therapeutic intervention in HD.

### Amyotrophic Lateral Sclerosis

ALS, also known as motor neuron disease, is characterized by the accumulation of inclusion bodies in the cytoplasm of motor neurons in the primary motor cortex, brainstem, and spinal cord, leading to progressive motor neuron loss (Ling et al. 2013; Abati et al. 2020; Obrador et al. 2021). The DNA- and RNA-binding protein TDP-43 is the main component of cytosolic aggregates in familial and sporadic ALS, and it has been found in more than 95% of cases. This is pivotal in treatment, as both loss of nuclear function and gain of cytoplasmic function are key pathogenic pathways (Huang et al. 2020; Wood et al. 2021).

**Fig. 5:**
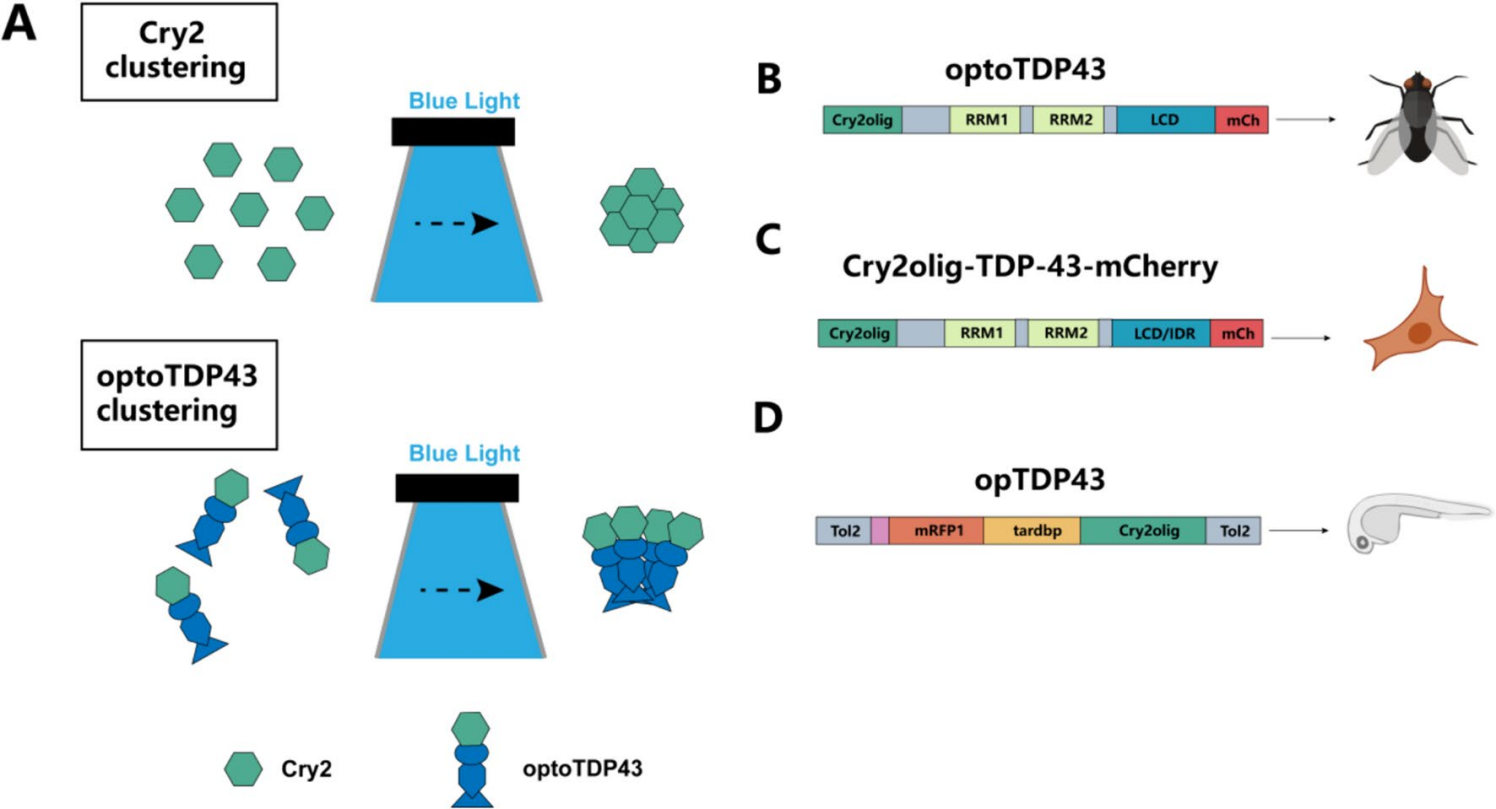
Model of TDP-43 proteinopathy induced by light. **A** Cry2 can aggregate under blue light. Light-inducible TDP-43 retinopathy induced by the Cry2olig photoreceptor and TDP-43 protein. **B** Diagram of optoTDP43 constructs and a model for its application in Drosophila. **C** Diagram of Cry2olig-TDP-43-mCherry constructs and a model for its application in human-corticoid nerve cells. **D** Diagram of opTDP-43 constructs and a model for its application in zebrafish larvae

The application of optogenetic techniques is beneficial for studying the process of TDP-43 aggregation and achieving precise regulation (Fig. 5). An optogenetic model of TDP-43 proteinopathy (optoTDP43) was developed in Drosophila (Fig. 5A) through light-induced mislocalization and aggregation of optoTDP43, increasing the functional toxicity of motor neurons in larval and adult Drosophila (Otte et al. 2020) (Fig. 5B). This method involved the use of channelrhodopsin in class IV dendritic arborization sensory neurons of Drosophila larvae, with a blue light control. Researchers then used behavioral experiments and calcium imaging combined with fluorescence recovery after photobleaching to demonstrate that TDP-43 localization is cell type dependent and dynamically changes during development. Cytoplasmic calcium plays a crucial role in regulating the distribution of TDP-43 between the nucleus and cytoplasm, and the calcium-calpain-A-importin α3 pathway is key for nucleocytoplasmic transport, leading to cytoplasmic TDP-43 accumulation in Drosophila neurons (Park et al. 2020).

The Cry2olig-TDP-43-mCherry expression vector, shown in Fig. 5C, allows precise control over the expression and localization of TDP-43 in human cortical-like neuronal cells using blue light. This model showed that the aberrant phase transition of cytoplasmic TDP-43 induced by optogenetic manipulation results in neurotoxicity, which can be prevented and reversed by oligonucleotide therapy involving the TDP-43 target sequence (Mann et al. 2019). Similarly, an optogenetic variant of TDP-43 (opTDP-43) (Fig. 5D) was employed in transparent zebrafish larvae. Short-term light stimulation led to the mislocalization of opTDP-43 in the cytoplasm of zebrafish larval spinal motor neurons, but it did not result in distinct aggregate formation. However, prolonged illumination eventually led to the accumulation of cytoplasmic opTDP-43 aggregates, which triggered the aggregation of nonoptogenetic TDP-43 and was accompanied by motor decline (Asakawa et al. 2020).

Furthermore, disturbances in stress granule (SG) dynamics have emerged as a significant contributing factor to the development of ALS. SGs are non-membrane-bound RNA‒protein granules that assemble through phase separation in response to cellular stress. Zhang P et al. expressed a light-sensitive protein called Opto-G3BP1 in cells and used blue light to activate its phase separation properties, which led to the formation of SGs. Chronic photogenetic induction of SGs is cytotoxic and progresses to cytoplasmic inclusions, consistent with the characteristic pathology of ALS (Zhang et al. 2019).

A neurophysiological three-dimensional model of ALS can also be established using optogenetic motor neurons, which are formed by 3D skeletal muscle bundles, induced pluripotent stem cell-derived and light-sensitive ChR2-induced motor neuron spheroids from a patient with sporadic ALS. The application of optogenetic technology enables accurate spatiotemporal control of neural activity and muscle contraction, enhances the functionality and applicability of this model, and provides a valuable platform for evaluating potential drug candidates and understanding pathogenesis (Osaki et al. 2018).

We summarized the current application of optogenetic technology in ALS, with the aspiration that this technology can offer convenience for mechanistic research and drug development (Table 4).

## Conclusion and Perspectives

Neurodegenerative diseases are generally associated with eight distinctive hallmarks, including pathological protein aggregation, disruptions in synaptic and neural network function, disturbances in protein homeostasis, irregularities in the cytoskeleton, alterations in energy homeostasis, DNA and RNA abnormalities, inflammation, and ultimately, the death of nerve cells (Wilson et al. 2023). The progression of neurodegenerative diseases is typically chronic, often resulting in patients experiencing a natural course that spans several years or even decades. Due to the intricate and varied nature of neural circuits, precise control over molecular targets holds exceptional significance within such an intricate system.

Optogenetic technology is a powerful tool for achieving precise regulation and its utilization is steadily expanding in the field of neurodegenerative disease research. It holds enormous potential for uncovering the complicated mechanisms behind neurodegenerative illnesses and developing original therapies. By integrating optogenetics with functional magnetic resonance imaging, researchers can selectively activate specific neural circuits while monitoring the real-time response of the entire brain, allowing for better mechanistic exploration (Asaad and Lee 2018). Furthermore, due to variations in the effects of different light-sensitive proteins, irradiation wavelengths, and irradiation durations, selecting the appropriate parameters for specific diseases is essential. For instance, frequent blue light activation of neurons expressing ChR2 in the substantia nigra pars compacta can continuously increase the activity of these neurons, potentially alleviating the dysfunction of the contralateral forepaw in PD rats. Conversely, yellow light activation of neurons expressing NpHR or similar inhibitory opsins can decrease the expression of tyrosine hydroxylase, resulting in defects in the adjusting steps of the contralateral forepaw (Heo et al. 2020). Apart from visible light, infrared and near-infrared light have unique advantages in optogenetic techniques. Compared with visible light, infrared and near-infrared light can penetrate biological tissues more deeply. This allows for the stimulation or inhibition of neurons at greater depths within the brain or other organs, expanding the reach of optogenetic interventions. Furthermore, infrared and near-infrared light are generally less damaging to tissues than are the higher-energy photons found in visible light (Kasatkina et al. 2022). This reduced phototoxicity is crucial for experiments requiring prolonged or repeated light exposure.

While holding groundbreaking promise, optogenetics confronts significant challenges. One of the primary limitations is the restricted penetration depth of light in biological tissues, which hinders the modulation of neurons located deep within the brain. Although controlling neurons near the brain’s surface is easier, targeting deep-seated neurons often requires surgically implanting optical fibers. This approach carries inherent risks associated with surgical procedures, such as infection and tissue damage. Moreover, the deeper the target neuronal location is, the more challenging it becomes to deliver light without substantially increasing its intensity, which could lead to tissue damage (Varady and Distel 2020).

To overcome the limitations of optogenetics, researchers are developing less invasive light delivery methods. For example, a novel step function opsin named SOUL, which has ultrahigh light sensitivity, has been designed to activate neurons in any mouse brain region via transcranial optical stimulation, regardless of location. It can also regulate neuronal spikes and reversibly induce oscillations in the cortex of rhesus monkeys by extradural light stimulation and a minimally invasive optogenetic technique has been developed to control the activity of neurons in mice and monkeys without implantation (Gong et al. 2020). At the same time, the advent of miniaturized fiber optic systems and wireless optogenetic devices has greatly improved the issue of invasiveness. A flexible, multimodal fiber probe was fabricated using a fiber drawing process, and it comprises metal electrodes for neural signal recording and a double-clad waveguide for optical stimulation. This design effectively combines electrical and optical modalities for advanced optogenetic applications and minimizes invasiveness in optogenetics by employing highly flexible, ultrathin, and biocompatible materials. It allows for gentle insertion into neural tissue, reducing physical disruption, minimizing the risk of inflammation and scarring, and enabling sustained, reliable neural interfacing with minimal impact on the surrounding brain tissue (Du et al. 2020). In addition, a novel ultraminiature, wireless, battery-free, and fully implantable optogenetic device has been developed. Its compact size allows for less invasive implantation through the wireless elimination of external wires or tethers, which reduces physical restrictions and potential tissue irritation, allowing for more natural animal behavior during experiments. The need for internal power sources such as batteries can be avoided by harnessing energy from external sources, such as radiofrequency fields, which can be bulky and require surgical replacement. These features make the device significantly less invasive than traditional optogenetic tools (Yang et al. 2021).

Another challenge is the thermal effects and potential tissue damage due to the absorption and scattering properties of light in biological tissues. When light, particularly from lasers transmitted through optical fibers, penetrates these tissues, a portion of the light energy is absorbed and converted into heat, leading to localized temperature increases and possibly tissue damage. Optogenetics can be combined with nanomaterials or through the development of highly light-sensitive proteins (Sardoiwala et al. 2019). They can minimize tissue damage and improve the modulation of deep brain neurons. For example, specific nanoparticles have been developed to convert extracranial infrared light into visible light within the brain, enabling noninvasive stimulation of ontogenetically modified neurons (Chen et al. 2018). In this way, thermal damage caused by fiber optic transmission through tissues can be avoided.

Furthermore, optogenetic control typically relies on viral vectors to transfect neurons with genes encoding opsins. Although this method enables specific neurons to respond to light, the use of viral vectors can sometimes trigger immune responses in the host organism, leading to inflammation or other complications. However, several strategies can reduce the immune response to viral vectors. For example, PEGylation uses synthetic polymers to shield surface antigens and employs lipid bilayer envelopes to protect viral vectors from antibody neutralization. In addition, the use of small-molecule proteasome inhibitors or the induction of peripheral immune tolerance to capsids and transgenes with drugs such as rapamycin achieves transient pharmacological immune modulation and alleviates the immune response caused by viral vectors. Furthermore, the shift from viral vectors to nonviral delivery methods, such as lipid nanoparticles, can reduce the potential for immune reactions (Sandbrink et al. 2023).

The emerging advancements in new technologies are progressively overcoming the limitations inherent in optogenetics. A brain–spine interface has recently been created to connect the brain to the spinal cord area associated with movement, which can assist chronic tetraplegic patients in standing, walking, climbing stairs, and even naturally controlling leg movement on challenging terrain (Lorach et al. 2023). However, the use of electrodes for BSI has natural drawbacks, such as mechanical mismatch between electrodes and neural tissue, which may affect the safety of brain tissue. Fortunately, a promising solution to address this challenge has been proposed by Ji et al. who successfully developed a stretchable optoelectronic integrated MEMS device based on optogenetic technology. This innovative device exhibits exceptional flexibility and a considerable degree of stretchability, enabling it to seamlessly adapt to brain tissue deformation or micromotion, effectively preventing any mechanical damage to delicate brain tissue. Moreover, the device demonstrates reliable long-term implantation capabilities, as it can harmonize with the mechanical stiffness of brain tissue while delivering high-precision synchronized optical stimulation and electrical recording. These impressive features render the device highly versatile, holding great potential for a wide array of applications in various types of neural interfaces (Ji et al. 2020).

This gives us ample reason to believe that, in future, optogenetic techniques will make significant contributions to the field of neurosciences, heralding a new era in medical science and therapeutic approaches.

## Data Availability

No datasets were generated or analysed during the current study.
